# The native state of prion protein (PrP) directly inhibits formation of PrP-amyloid fibrils in vitro

**DOI:** 10.1038/s41598-017-00710-x

**Published:** 2017-04-03

**Authors:** Ryo P. Honda, Kazuo Kuwata

**Affiliations:** 1 Department of Molecular Pathobiochemistry, Graduate School of Medicine, Gifu University, 1-1 Yanagido, Gifu, 501-1193 Japan; 2 United Graduate School of Drug Discovery and Medical Information Sciences, Gifu University, 1-1 Yanagido, Gifu, 501-1193 Japan; 3 Department of Gene and Development, Graduate School of Medicine, Gifu University, 1-1 Yanagido, Gifu, 501-1193 Japan

## Abstract

The conversion of globular proteins into amyloid fibrils is associated with a wide variety of human diseases. One example is the prion protein (PrP), which adopts an α-helical structure in the native state but its amyloid form is implicated in the pathogenesis of prion diseases. Previous evidence has suggested that destabilization of the native state promotes amyloid formation, but the underlying mechanism remains unknown. In this study, we report that the native state of PrP serves as a potent inhibitor in the formation of PrP amyloid fibrils. By monitoring the time courses of thioflavin T fluorescence, the kinetics of amyloid formation was studied *in vitro* under various concentrations of pre-formed amyloid, monomer, and denaturant. Quantitative analysis of the kinetic data using various models of enzyme kinetics suggested that the native state of PrP is either an uncompetitive or noncompetitive inhibitor of amyloid formation. This study highlights the significant role of the native state in inhibiting amyloid formation, which provides new insights into the pathogenesis of misfolding diseases.

## Introduction

The formation of amyloid fibrils or amyloid-like aggregates is one characteristic feature in a variety of human diseases^1^. A wide range of such misfolding diseases is associated with the conversion of globular proteins into amyloid fibrils. Examples include hemodialysis-related amyloidosis, amyotrophic lateral sclerosis, and prion diseases. The kinetics of amyloid formation has been extensively studied to understand the fundamental mechanism underlying misfolding diseases^2–9^. It is well established that globular proteins have an increased propensity to amyloid formation under solution conditions where the native structure is destabilized by pH, high temperature, and the addition of chaotropic agents such as urea and guanidine hydrochloride (GuHCl)^10^. However, it is not well understood how destabilization of the native structure is related to amyloid formation. This motivated us to investigate the kinetics of amyloid formation by prion protein (PrP). PrP adopts an α-helical structure in the native state^11^ but has the ability to form amyloid fibrils^12–15^ and amyloid-like aggregates^16–19^ under conditions that destabilize the native form. The amyloid forms of PrP have been implicated in the pathogenesis of prion diseases^20, 21^.

## Results and Discussion

### A systematic investigation of amyloid formation *in vitro*

**Figure 1:**
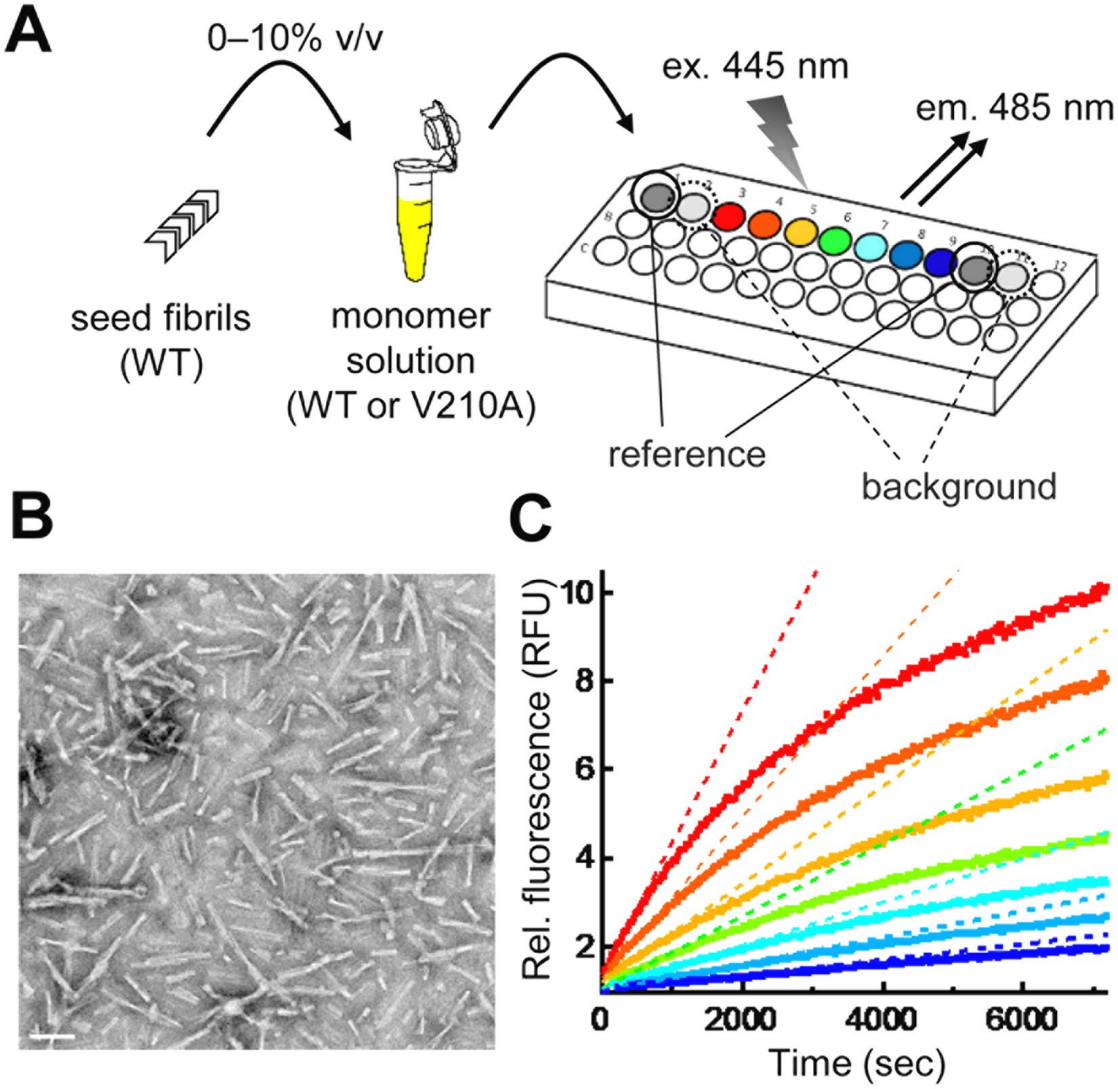
(**A**) A schematic representation of seeded growth experiments. (**B**) Transmission electron microscopy images of seed fibrils (scale bar, 100 nm). (**C**) A representative result of the seeded growth experiment. The growth reaction was examined at a fixed concentration of seed fibrils (2.5%) and GuHCl (3.0 M) by varying the monomer concentration from 5 to 300 µM (from bottom to top). The dotted lines represent the linear fits to the initial increase in fluorescence.

In this section, we will briefly outline our experimental strategy for investigating amyloid formation by PrP. We performed a series of seeded growth experiments in which pre-formed amyloid fibrils (“seeds”) were added to a solution containing a bacterially-expressed recombinant PrP (rPrP), and their growth under a quiescent condition was continuously monitored at 37 °C through the change in thioflavin T (ThT) fluorescence (Fig. 1A and B). The seed fibrils were generated from rPrP using serial protein misfolding cyclic amplification (PMCA)^22^. We employed a microtiter plate method to rapidly examine a wide variety of different conditions (Fig. 1A). A total of 264 different conditions were studied by systematically varying the concentrations of seed fibrils, monomer, and GuHCl. To facilitate direct comparison of the results, the measured fluorescence was normalized to that of a reference sample containing 2.5% seed fibrils and the same concentration of GuHCl but no monomer.

Figure 1C shows a representative result when amyloid formation was examined at a fixed concentration of seed fibrils (2.5%) and GuHCl (3.0 M) by varying the concentration of monomer. The addition of seed fibrils completely eliminated the lag phase and we observed an exponential increase in ThT fluorescence. This overall behavior is similar to the growth reaction observed in other amyloid systems^23^, indicating that the growth of the pre-existing seed is the dominant process in our system.

### Effects of varying the seed concentration on amyloid growth

We performed the first set of seeded growth experiments at a fixed monomer concentration (18 µM) by varying the seed concentration from 0 to 10% and the GuHCl concentration from 1.3 to 5.0 M. The structure of seed fibrils was essentially identical for the entire concentration range of GuHCl, as judged by far UV circular dichroism (CD) (Figure S1). In addition, the biases associated with ThT fluorescence^24^ are negligible under these conditions, as the rate of amyloid growth is almost identical in the presence or absence of ThT (Figure S2 and SI Text 2). No significant growth of amyloid fibril was observed below 1.3 M GuHCl at least up to three hours (data not shown).

**Figure 2:**
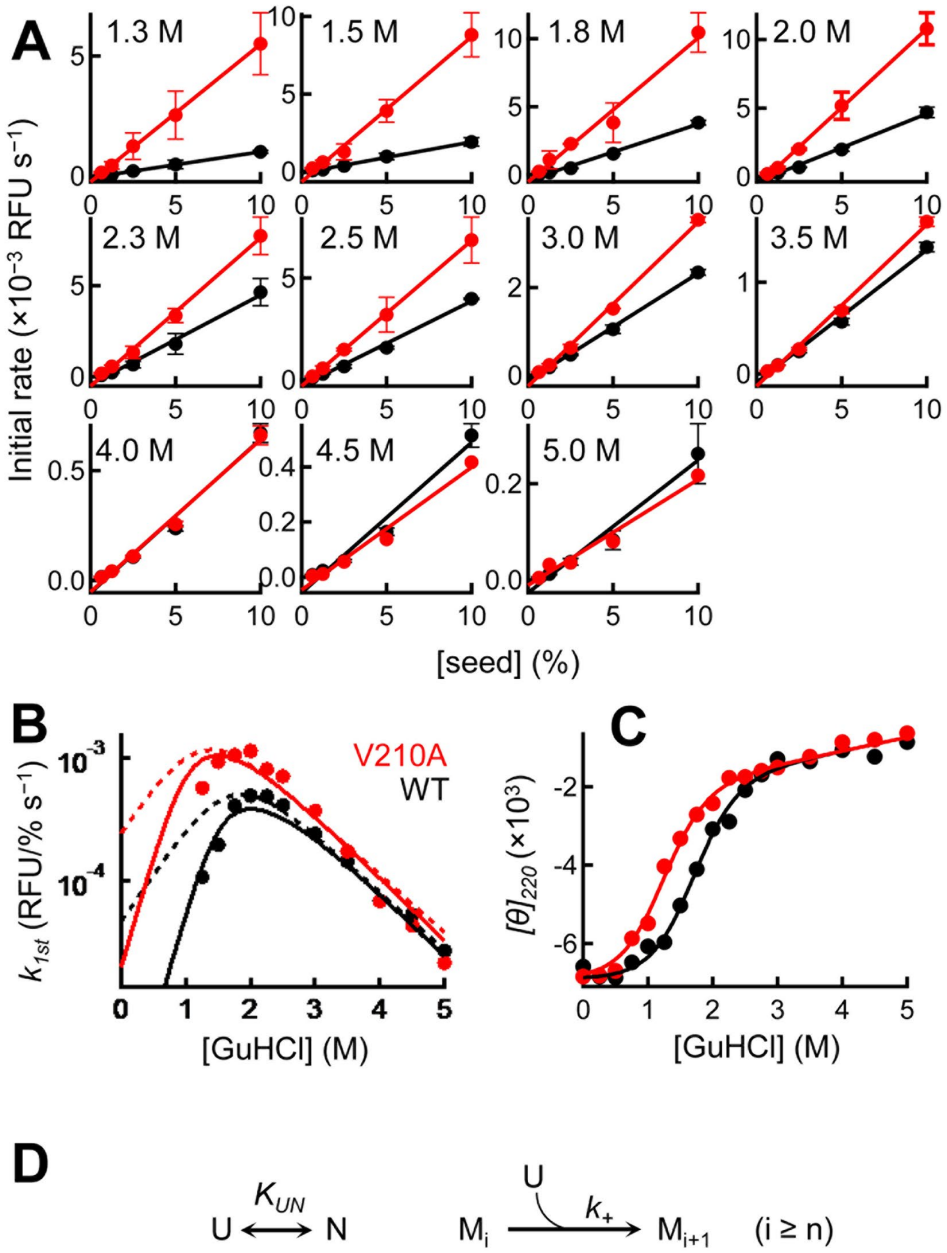
Effects of varying the seed concentration on amyloid growth. (**A**) Initial rates of the growth reaction (WT [black] and V210A [red]) as a function of the seed concentration. The GuHCl concentrations in the reaction mixtures are shown in the top left of each images. The monomer concentration was 18 µM in all experiments. (**B**) Pseudo-first order rate constants of WT (black) and V210A (red circles) as a function of the GuHCl concentration. The dotted and solid lines represent least-squares fits to the one-step (Fig. 2D) and the native-state inhibition models (Fig. 5), respectively. (**C**) Equilibrium unfolding curves of monomeric WT (black) and V210A (red) obtained by CD. Solid lines represent a least-square fits to the two-state unfolding model. (**D**) One-step reaction model for amyloid growth. M_i_ represents an amyloid fibril with the polymerization degree of *i* and *n* is the size of the critical nucleus. U and N is the unfolded and native state of PrP, respectively.

As shown in Figure S3, nearly exponential growth of seed fibrils was observed in all 55 cases examined. To quantitatively evaluate the growth reaction, we measured the rates of formation of the first few percent of amyloid fibrils (initial rate) and displayed them as black circles in Fig. 2A. Over the concentration range evaluated, the initial rates were directly proportional to the concentration of the added seed. Therefore, the amyloid growth of PrP is simply described by the pseudo-first order kinetics with respect to the seed concentration.

The black circles in Fig. 2B show the pseudo-first order rate constant (*k*_*1st*_) under various concentrations of GuHCl. The log(*k*_*1st*_) vs. [GuHCl] plot displayed a bell-shaped profile with a peak around 2.3 M, and a wide linear range was found between 2.5 and 5.0 M. Interestingly, far UV CD experiments revealed that this linear range corresponds to the concentration at which monomeric PrP is completely unfolded (black circles in﻿ Fig. 2C). This result implied that, at least when the substrate monomer is unfolded, amyloid growth follows the transition state theory in chemical kinetics, in which the logarithm of the rate constant is linearly correlated with the denaturant concentration^25^. As the concentration of GuHCl decreases from 2.5 to 1.3 M, the fraction of folded proteins increases (Fig. 2C), and log(*k*_*1st*_) starts to deviate from the linear dependence (Fig. 2B). This type of bell-shaped curve resembles the “rollover” in protein folding^25^, and it was previously reported in the growth reaction of insulin amyloid^7^.

The relation between rollover and the stability of the native state was further investigated using a single-site variant of PrP (V210A) as the substrate monomer. The V210 residue is located in the hydrophobic core of PrP, and the replacement of valine with alanine lowered the stability of the native state by 0.87 kcal/mol (Fig. 2C). We performed equivalent experiments with this variant and measured the initial rates under various concentrations of seed fibrils and GuHCl (Fig. 2A and Figure S4). As shown in Fig. 2B, rollover was less pronounced in this variant compared with that in the wild-type (WT), indicating that the stability of the native state (“N”) is a key determinant of the observed behavior.

Based on these observations, we first hypothesized that rollover stems from a decreased population of a non-native state under the stabilizing conditions that can be directly converted into amyloid fibrils^26^. Although it remains controversial whether a partially or a completely unfolded state is actually involved^27–32^, we considered the completely unfolded state (“U”) as the direct precursor of amyloid fibrils in this particular case. This is partly because no partially unfolded state was detected in the GuHCl unfolding experiment (Fig. 2C), where the unfolding curves at all wavelengths can be globally fitted to the two-state unfolding model (Figure S5). Previous hydrogen/deuterium exchange and spectroscopic experiments also showed that most of the population of PrP is in either the N or U state at neutral pH^33–35^. Furthermore, a large difference between the α-helix structure of PrP and the parallel-in-register β-sheet structure of the amyloid form^11, 36–38^ implies that the native structure requires complete unfolding prior to the conversion. In addition to these experimental evidences, the rollover curve with a peak at around the denaturant midpoint can be explained without assuming any partially unfolded state, as described below. We therefore first tested a simple one-step reaction model in which the U state serves as a direct precursor of amyloid growth (Fig. 2D). It should be noted that rapid equilibrium between the N and U states is assumed in this model and the rate-limiting step is the bimolecular reaction. This was verified by the fact that the exchange rate between the N and U state is extremely rapid^39^, which is estimated to be 1000 s^−1^ at 37 °C in our ongoing folding/unfolding experiments (data not shown).

Following Oosawa’s treatment^40^, we solved the rate and equilibrium equations of the one-step model to obtain the time evolution of the mass concentration of amyloid fibrils as follow:

1 $$\frac{d}{dt}(\sum _{i=n}^{\infty }i{{\rm{\lambda }}}_{i}(t))={k}_{+}{f}^{U}\,{\rm{\lambda }}({\rm{t}})\sum _{i=n}^{\infty }{{\rm{\lambda }}}_{i}(t)$$

where *f* ^*U*^ is the fraction of the U state; *λ(t)* and $\sum _{i=n}^{\infty }{{\rm{\lambda }}}_{i}(t)$ are the number concentrations of monomer and amyloid fibrils, respectively. This rate equation implies that the amyloid growth behaves similarly as a second-order reaction with the pseudo-order first rate constant of *k*_+_ *f* ^*U*^ (*k*_*1st*_ = *k*_+_ *f* ^*U*^). This simple one-step model can qualitatively explain why the log(*k*_*1st*_) vs. [GuHCl] plot yields the rollover curve. This is because the denaturant has opposing effects on *k* ^+^ and *f* ^*U*^, in which *k* ^+^ is negatively dependent on [GuHCl] (because the transition state between U and M_i+1_ is more structured than U) while *f* ^*U*^ is positively dependent on [GuHCl]. However, a detailed kinetic analysis revealed that this simple model is unable to quantitatively reproduce the rollover curves in either WT nor V210A (dotted lines in Fig. 2B), where it significantly overestimates the growth rates at less than 2 M GuHCl. Therefore, the one-step model should be modified by including an inhibitory effect which reduces the growth rates at less than 2 M GuHCl.

### Effects of varying the concentration of the U state on amyloid growth

**Figure 3:**
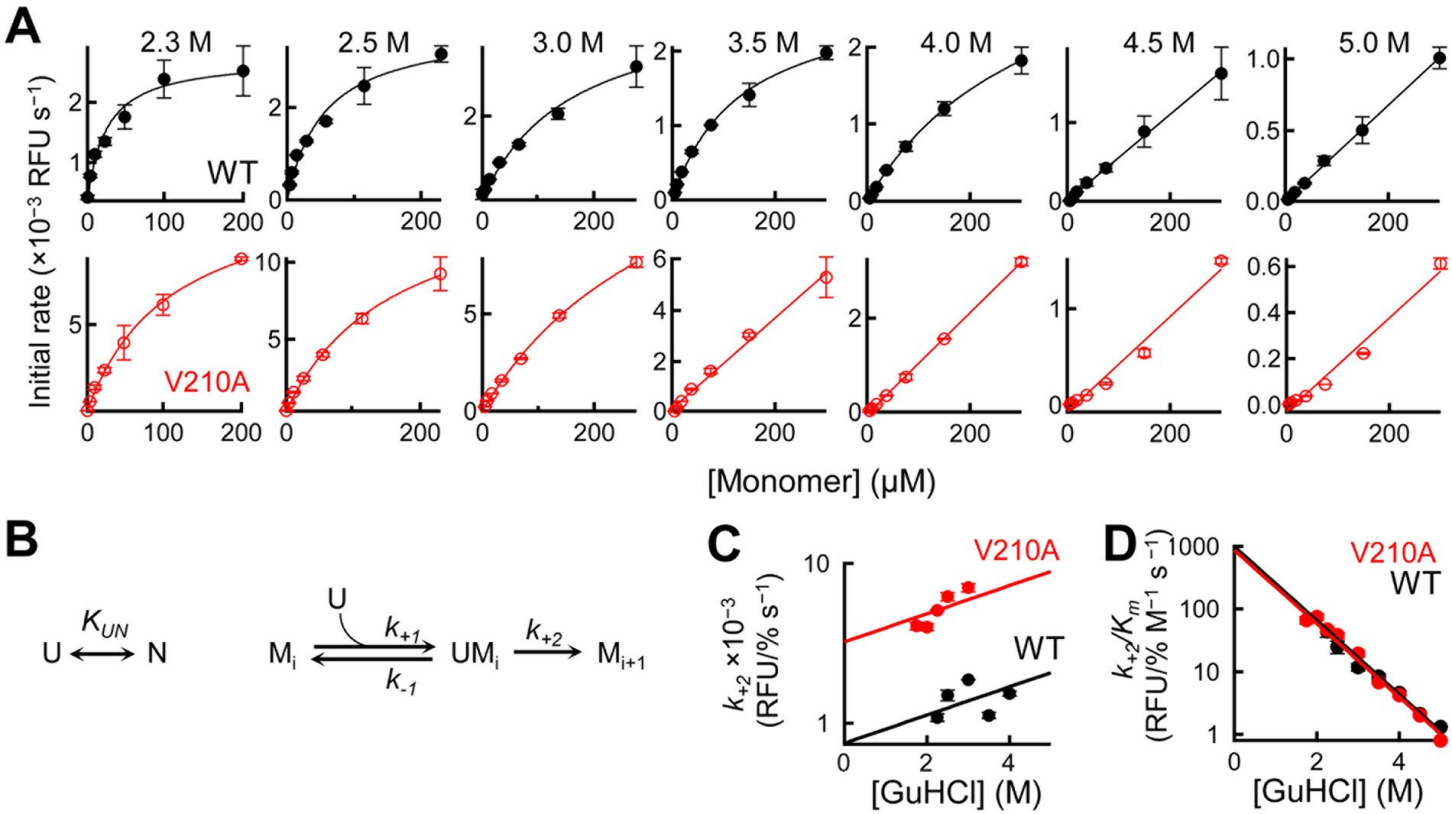
Effects of varying the concentration of the U state on amyloid growth. (**A**) Initial growth rates as the function of the total concentration of monomers (WT [top row] and V210A [bottom row]). The growth reaction was seeded with 2.5% WT amyloid. The solid lines are least-squares fits to the Michaelis–Menten function (Equation 2). (**B**) Two-step reaction model for amyloid growth. (**C,D**) Elementary constants derived from Fig. 3A as a function of the concentration of GuHCl [*k*_+*2*_ (**C**) and *k*_+*2*_/*K*_*m*_ (**D**)]. Note: 1 relative fluorescence unit (RFU)/% s^−1^ ≈1.2 × 10^3^  s^−1^ (SI text 5).

To further understand the relation between amyloid growth and stability of PrP, we next performed a seeded growth experiment at a fixed concentration of seed fibrils (2.5%) by varying the concentration of monomer from 5 to 300 µM. In this experiment, we first focused on the high concentration range of GuHCl (2.3–5.0 M), where the N state is essentially absent (Fig. 2C), in order to directly evaluate the effect of the U state on amyloid growth. As shown in Fig. 3A, while monitoring amyloid growth in the presence of 2.3–3.5 M of GuHCl, a saturation kinetics was observed for both WT and V210A (see also Figures S6 and S7); at low concentrations of the substrate monomer, the growth rate increased linearly, but it reached a maximum value at the high concentrations. We eliminated the possibilities of the oligomerization of PrP (Figure S8 and SI Text 3) or the slow binding of ThT (Figure S9 and SI Text 4) as the cause of saturation kinetics, by measuring dynamic right scattering (DLS), size exclusion chromatography (SEC), and *ex situ* ThT fluorescence. Indeed, saturation kinetics has been reported for a number of different amyloids, including Sup35^41, 42^, insulin^43^, α-lactalbumin^44^, α-synuclein^23^, and S6^45^, suggesting that this phenomenon is a general feature in the amyloid growth reaction.

To extend the one-step model (Fig. 2D) to cover the saturation kinetics, we next considered a reaction that involves a transient complex consisting of U state and amyloid fibrils (UM_i_) (Fig. 3B). This type of two-step model has been proposed by various authors on the basis of nuclear magnetic resonance^46^, atomic force microscopy^47, 48^, fluorescence microscopy experiments^49, 50^. This is the analog of the Michaelis–Menten scheme in enzyme kinetics^51^, and the initial rate under steady-state approximation is

2 $$\begin{array}{c}{(\frac{d}{dt}\sum _{i=n}^{\infty }i{{\rm{\lambda }}}_{i}(t))}_{t=0}=\,\frac{{k}_{+2}{f}^{U}{\rm{\lambda }}(0)}{{K}_{m}+{f}^{U}{\rm{\lambda }}(0)}\sum _{i=n}^{\infty }{{\rm{\lambda }}}_{i}(0)\end{array}$$

where *K*_*m*_ is the Michaelis constant and is equal to *(k*_*−1*_ + *k*_+*2*_ *)/k*_+*1*_. When the concentration of the U state (*f* ^*U*^ *λ(0)*) is larger than *K*_*m*_, the reaction rate tends to reach a limiting value, which is consistent with our current observations.

Relying on the two-step model, we then calculated the elementary constants using the least-square fitting method, and displayed them in Fig. 3C and D (see also Tables S1 and S2). One notable feature in the set of elementary constants is that the rate constant of structural conversion (*k*_+*2*_) is in the range of 1–10 × 10^−3^ relative fluorescence unit (RFU)/% s^−1^ (Fig. 3C). Given that 1 RFU/% s^−1^ is roughly equal to 1.2 × 10^3^ s^−1^ (SI Text 5), *k*_+*2*_ ≈ 1–10 s^−1^, which is in good agreement with the folding rate constant of β-sheet-rich proteins, such as the β2-microglobulin (1–10 s^−1^) and β-lactoglobulin (0.1–1 s^−1^)^52, 53^. This agreement further validates the two-step model, because *k*_+*2*_ is the refolding rate constant from the U state to the β-sheet amyloid structure on the surface of amyloid fibrils (Fig. 3B).

The comparison between WT and V210A showed that the V210A mutation leads to a fourfold increase in *k*_+*2*_ (Fig. 3C), indicating that the V210 residue is involved in the structural conversion. However, the increase in *k*_+*2*_ was exactly compensated by an increase in *K*_*m*_ and, consequently, we observed a common value of *k*_+*2*_/*K*_*m*_ between V210A and WT (Fig. 3D). A similar trend of *k*_+*2*_/*K*_*m*_ was also observed in our preliminary experiment, in which *k*_+*2*_ at pH 7.4 was significantly higher than that at pH 6.0 but *k*_+*2*_/*K*_*m*_ was equal between the two conditions (data not shown). These results imply that *k*_+*2*_/*K*_*m*_ itself is an elementary constant in the reaction kinetics that represents a general feature of amyloid growth. One possible scenario to explain this finding is to assume the Briggs–Haldane mechanism^51^, in which *k*_+*2*_/*K*_*m*_ is equal to the rate constant of diffusion-controlled encounter, namely *k*_+*1*_, and thus is not strongly affected by mutations and pH. In fact, the linear extrapolation of log(*k*_+*2*_/*K*_*m*_) to 0 M GuHCl yields a value of 1.2 × 10^6^ M^−1^ s^−1^ (Fig. 3D), which is very close to the diffusion-controlled rate of 10^7^–10^8^ M^−1^ s^−1^ ^51^. The small difference between the two values can be explained in terms of a small free-energy barrier separating the U state from the encounter complex (UM_i_). Therefore, our results imply that the amyloid growth follows the Briggs–Haldane model, where U state initially binds to amyloid fibrils with a rate close to the diffusion limit, and subsequently undergoes a slow structural conversion with a rate of 1–10 s^−1^.

### An inhibitory effect of the N state on amyloid growth

**Figure 4:**
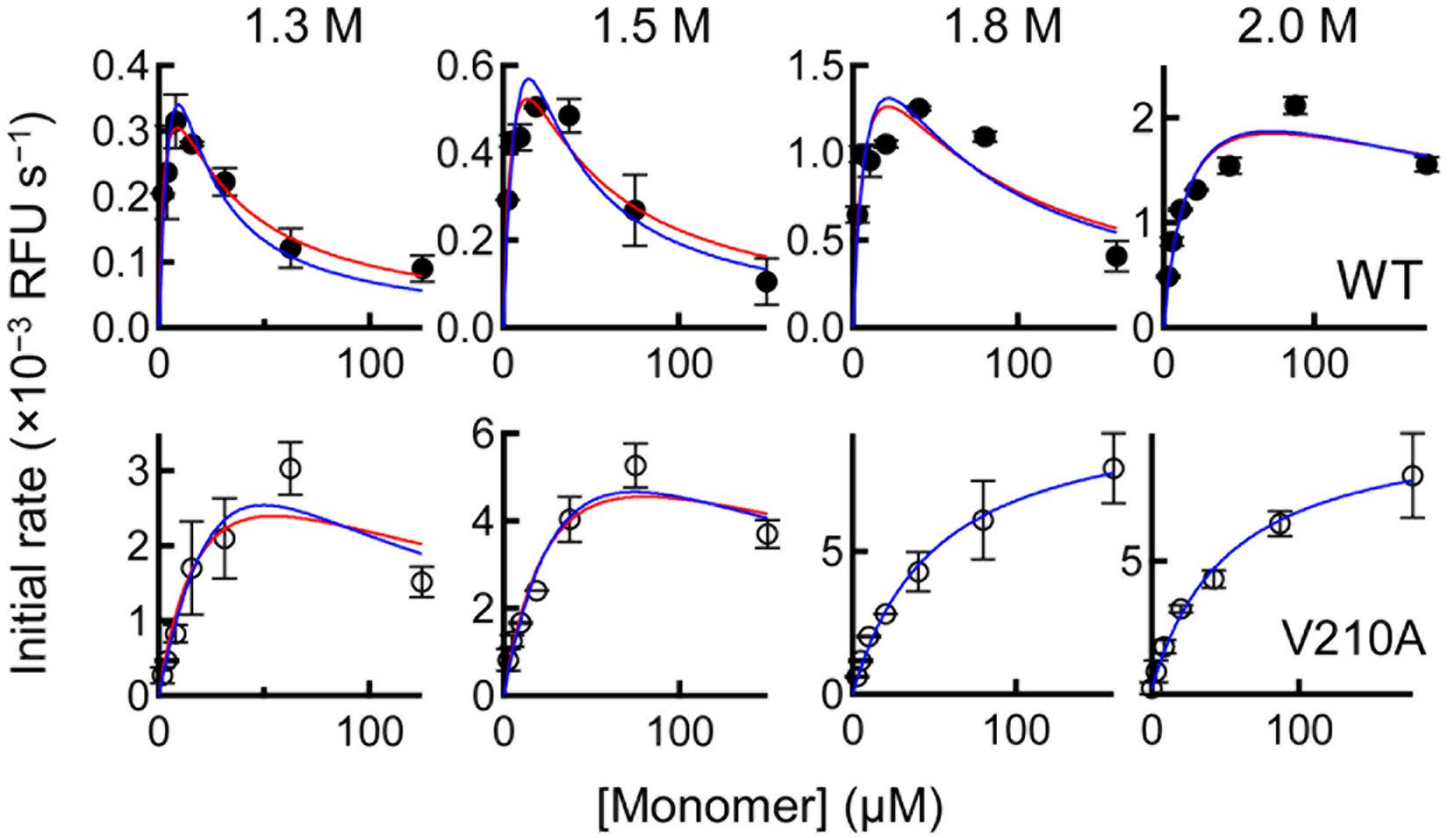
Effects of varying the concentration of the N and U state on amyloid growth. Initial rates of amyloid growth as a function of total concentration of monomers (WT [top row] and V210A [bottom row]. The growth reaction was seeded with 2.5% of WT amyloid. The solid lines are least-squares fits to Equation 3 (red, uncompetitive inhibition) or 4 (blue, noncompetitive inhibition).

Having established the relation between amyloid growth and the U state, we next sought to determine how the N state affects the kinetics of amyloid growth. To this end, we performed a seeded growth experiment in the lower concentration range of GuHCl (1.3–2.0 M), where both the N and U states are populated (Fig. 2C), by varying the total concentration of the monomers. Surprisingly, under these conditions the reaction rate first rose to a maximum value and then declined as the total monomer concentration increased (Fig. 4, Figures S5 and S6). This “up-and-down” behavior called for further modification of the two-step model (Fig. 3B and Equation 2) because the first derivative of Eq. 2 with respect to monomer concentration is always positive.

We first invoked the substrate-inhibition mechanism in enzyme kinetics, in which the substrate also acts as an inhibitor of enzyme^51^. This mechanism can cause a decline of the reaction rate when the substrate is present at a high concentration. However, this is not the case in amyloid growth because the decline of reaction rate is less pronounced in destabilizing conditions which favor the formation of the substrate (U state), such as in V210A (bottom﻿ panels in ﻿Fi﻿g. 4) and at the high concentration range of GuHCl (Fig. 3A). This result indicates that the inhibitor of amyloid growth is not the U state but a more structured state which can be populated under stabilizing conditions.

**Figure 5:**
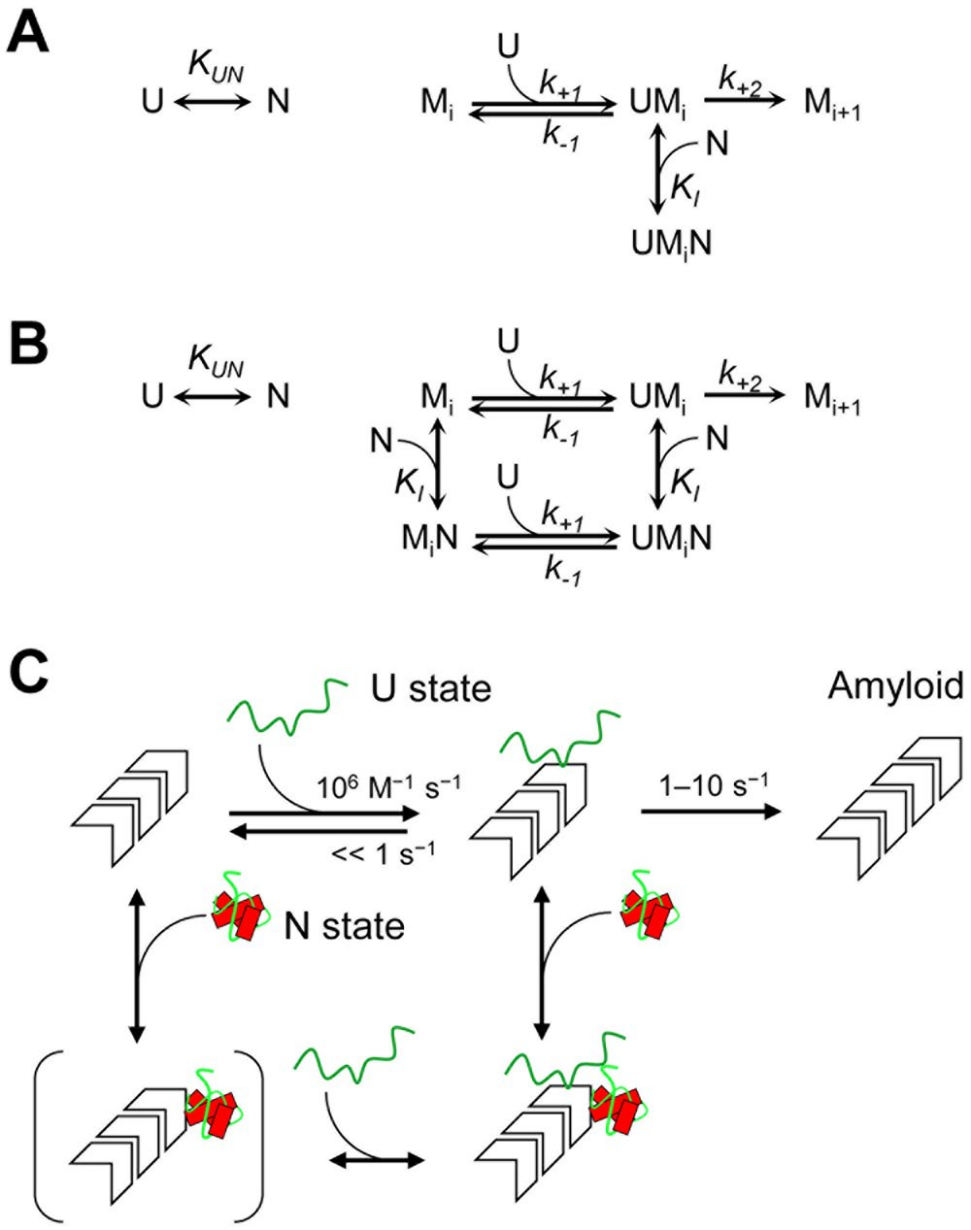
(**A**) Uncompetitive inhibition model (Equation 3). (**B**) Noncompetitive inhibition model (Equation 4). (**C**) A schematic representation of native-state inhibition model. The unfolded (U) state of PrP serves as the direct precursor of amyloid fibrils, whereas the native (N) state inhibits the amyloid formation thorough uncompetitive or noncompetitive inhibition.

Because there was no detectable partially unfolded state or oligomeric state in our GuHCl-unfolding (Fig. 2D) and DLS/SEC experiments (Figure S7), we thought that the native state (N) might be an inhibitor of amyloid growth. After testing several alternative models, we developed two models that include the inihibitory effect of N state and reproduce the “up-and-down” behavior of amyloid growth (Fig. 5). The first model assumes that N state interacts with a complex consisting of U state and amyloid fibrils, but has no direct interaction with amyloid fibrils itself (uncompetitive inhibition, Fig. 5A). The second model assumes that the N state interacts with the amyloid fibrils regardless of whether the U state is already bound (noncompetitive inhibition, Fig. 5B). Solving the equilibrium and rate equations of these schemes gives the initial rates of the reaction

3 $${(\frac{d}{dt}\sum _{i=n}^{\infty }i{{\rm{\lambda }}}_{i}(t))}_{t=0}=\,\frac{{k}_{+2}{f}^{U}{\rm{\lambda }}(0)}{{K}_{m}+{f}^{U}{\rm{\lambda }}(0)+\frac{{f}^{N}{f}^{U}}{{K}_{I}}\lambda {(0)}^{2}}\sum _{i=n}^{\infty }{{\rm{\lambda }}}_{i}(0)$$

for uncompetitive inhibition and

4 $${(\frac{d}{dt}\sum _{i=n}^{\infty }i{{\rm{\lambda }}}_{i}(t))}_{t=0}=\,\frac{{k}_{+2}{f}^{U}{\rm{\lambda }}(0)}{{K}_{m}+({f}^{U}+{f}^{N}\frac{{K}_{m}}{{K}_{I}}){\rm{\lambda }}(0)+\frac{{f}^{N}{f}^{U}}{{K}_{I}}\lambda {(0)}^{2}}\sum _{i=n}^{\infty }{{\rm{\lambda }}}_{i}(0)$$

for noncompetitive inhibition, where the *f* ^*N*^ is the fraction of the N state and thus *f* ^*N*^ *λ(0)* is the concentration of the N state at time zero. These equations can be easily derived from the basic equations in enzyme kinetics^51^ by substituting the *f* ^*N*^ *λ(0)* for the inhibitor concentration and *f* ^*U*^ *λ(0)* for the substrate concentration.

Unfortunately, we cannot rule out either inhibition models, as the two rate equations include the completely same quadratic term in their denominators (*f* ^*N*^ *f* ^*U*^ *λ(0)* ^2^ */K*_*I*_). This results in a similar deceleration curve in the initial rates vs. [monomer] plot (Fig. 4) and hence makes it challenging to distinguish between the two models by reaction rates only. Although such ambiguity remains, it is clear that either the uncompetitive or noncompetitive inhibition model can reproduce the observed reaction rate with good quality (Fig. 4). In addition, either models can also reproduce the log(*k*_*1st*_) vs. [GuHCl] plot in the first set of experiments, particularly at the low [GuHCl] range (Fig. 2B). We therefore concluded that the N state inhibits amyloid growth through either an uncompetitive or noncompetitive inhibition mechanism. It should be noted that a competitive model^51^, in which the N state inhibits the interaction between the U state and amyloid fibrils, is unable to reproduce the deceleration curve, because the rate equation lacks a quadratic term in its denominator.

Our native-state inhibition model suggests that an amyloid fibril (and/or its complex with the U state) has two distinct binding sites for the U and N states, through which the former is associated with the initiation of amyloid formation and the latter is associated with its inhibition (Fig. 5C). The presence of more than one binding site is not surprising, as multiple peptides derived from PrP can be converted to an amyloid fibril with an in-register β-structure^54–57^. This fact implies that amyloid fibrils from full-length PrP contains multiple regions each having the ability to interact with monomeric PrP. Indeed, a previous study by Horiuchi *et al*. showed that heterologous PrP can inhibit the pathogenic conversion of homologous PrP in a noncompetitive manner^58, 59^. Although our study revealed an interference between homologous but structurally different PrPs, these two studies consistently demonstrate the presence of two functionally different sites (initiation and inhibition) in an amyloid-like aggregate.

**Figure 6:**
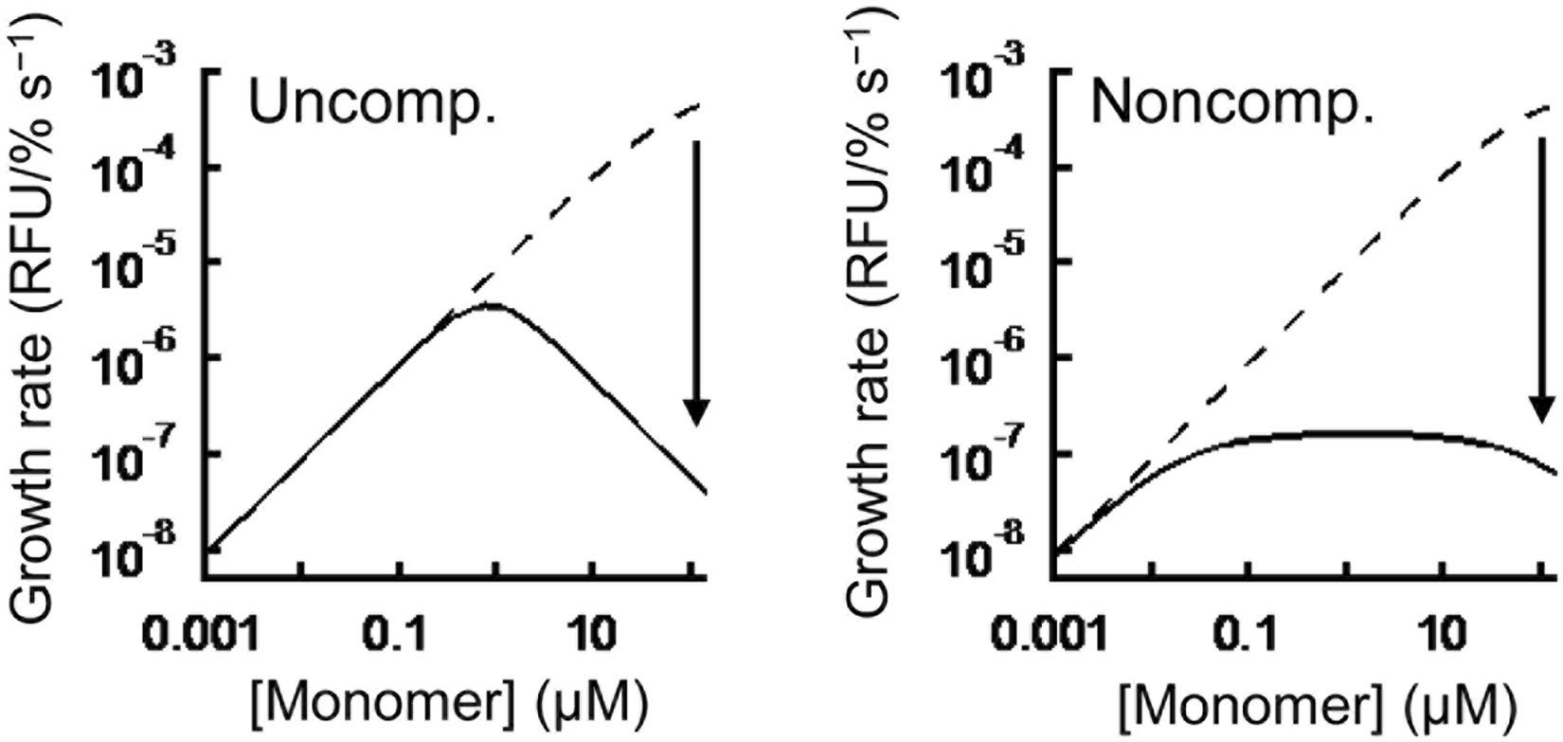
Native-state inhibition under a physiologically relevant condition. Predicted growth rate at 0 M GuHCl as a function of the monomer concentration (uncompetitive [left panel] and noncompetitive inhibition models [right panel]). The solid and dotted lines are the growth rates in the presence or absence of native-state inhibition, respectively.

An interesting question is whether native-state inhibition could also occur *in vivo*. Because our seed growth experiments were performed in the presence of GuHCl, we calculated the rate of amyloid growth in a GuHCl-free condition by extrapolating the obtained elementary constants (Fig. 6, Tables S1 and S2). Under this more physiological condition, the effect of native-state inhibition is dependent on the total concentration of monomers. In the low concentration range up to 0.1–1 µM, the inhibitory effect is minimal or absent, but the growth rate is dramatically decreased by 1–3 order of magnitudes in the high concentration range exceeding 0.1–1 μM. Although the physiological concentration of PrP in neural tissue is unknown at this time, it should be higher than that of the cerebrospinal fluid (0.01 μM)^60^. We therefore thought that native-state inhibition can also occur *in vivo* when the concentration of soluble PrP exceeds 0.1–1 µM.

### Therapeutic implications

This study supports the suggestion by Yuan *et al*. that the bacterially-expressed recombinant PrP (rPrP) is useful for the treatment of prion disease^61^. Yuan *et al*. employed a PMCA method to show that rPrP inhibits the production of proteinase K-resistant form with an IC_50_ of 60 nM. This result is excellently consistent with the current study, in which rPrP inhibits the production of amyloid fibrils with a submicromolar range under the physiologically-relevant condition (Fig. 6). According to the native-state inhibition model (Fig. 5), this inhibitory effect can be further enhanced by stabilizing the N state of rPrP, because the population of the substrate form (U state) is decreased and that of the inhibitory form (N state) is increased simultaneously. Therefore, combination therapy using a stabilizing ligand^62, 63^ in conjunction with a stable variant of rPrP, such as a disulfide-linked variant^64^ and V209M^65^, might be a promising strategy for treating prion diseases.

## Methods

### Materials

ThT (Wako), GuHCl (Nacalai Tesque, specially prepared reagent) and 2-(N-morpholino)ethanesulfonic acid (MES, Nacalai Tesque, specially prepared reagent) were dissolved with deionized water and passed through a 0.2 µm filter (Sartorius, Minisart® plus 17823 K) before use. The concentration of ThT was determined by a UV-Vis spectroscopy (Shimadzu, UV-2550) using the extinction coefficient of 36,000 M^−1^ cm^−1^ at 412 nm, and that of GuHCl was determined by a refractometer using the previously reported values^66^. The pH of the MES solution was adjusted by adding 6N sodium hydroxide so that the final pH of the reaction mixture was 6.0.

### Expression and purification of human prion proteins

A plasmid encoding the full-length human prion protein (hPrP[23–230]) was kindly gifted by Dr. Kurt Wüthrich (ETH Zurich, Switzerland). A single mutation (V210A) was introduced into the plasmid using a PrimeSTAR Mutagenesis Basal Kit (Takara), and the presence of the mutation was confirmed by DNA sequence. The resulting plasmids [hPrP(23–230) and hPrP(23–230, V210A)] were expressed and purified according to a previously published protocol^62^ with the following modifications. After the completion of oxidation, a solution containing 2 mg/mL protein and 8 M urea was 2.7-fold diluted with a buffer (10 mM Tris, 100 mM sodium phosphate, pH 8). Then the protein solution was supplemented with thrombin (Wako) at a final concentration of 10 units/mg and incubated at 25 °C for 16 h to cleave the His-tag. Undigested proteins and other impurities were removed by reverse-phase HPLC with a COSMOSIL 5C4-AR-300 column (Nacalai Tesque). The eluted solution was dried using a freeze dryer (EYELA, FDY-2200) and dialyzed at 4 °C against deionized water. The dialyzed protein was lyophilized again and stored at −30 °C until use. The purity of the proteins was greater than 95% as judged by non-reduced SDS-PAGE. The matrix-assisted laser desorption ionization time-of-flight mass spectrometer was used to confirm the molecular weight of the proteins. The lyophilized protein was dissolved with 6 M GuHCl prior to use, at a protein concentration of 600–800 µM, to remove any possible aggregates. The protein concentration was determined by the UV-Vis spectroscopy using an extinction coefficient of 56590 M^−1^ cm^−1^ at 280 nm.

### Preparation of a seed solution

Seed fibrils were prepared in a 6 mL reaction mixture containing 50 µM hPrP(23–230), 20 mM MES (pH 6), and 3 M GuHCl. Thirty second ultrasonic pulses with an interval of 9.5 min were repeatedly applied to the reaction mixture at 37 °C using a programmable sonicator equipped with a microplate horn (Misonix, QSONICA 431MPX). The amplitude of the sonicator was set to 10%. Fibril formation was followed by *ex situ* ThT measurements (see Supplemental experimental procedures), and allowed to proceed until ThT fluorescence reached a maximum value. The seed solution was dispensed into small volume aliquots (30 µL), frozen with liquid nitrogen, and stored at –80 °C until use. The same seed preparation was used throughout this study. Before use, the seed solutions were thawed on ice and sonicated for 30 s at 10 °C using the aforementioned instrument.

### Analysis of amyloid growth

Amyloid growth was examined at 37 °C in 20 mM MES (pH 6), 50 µM ThT in the presence of various concentrations of GuHCl, seed fibrils, and monomers (WT or V210A). The reaction was initiated by mixing a monomer solution (pre-warmed at 37 °C) with various amounts of the seed solution. The reaction mixture of 100 µL was immediately transferred to a pre-warmed, flat-bottom 96-well plate (AS ONE, Cat. No. 1–6776–03) and tightly sealed with transparent film (WATSON, Cat. No. 547-KTS-HCP). The time courses of the ThT fluorescence at 485 nm (excitation at 445 nm) were measured by top-reading under a quiescent condition using a plate reader (TECAN, infinite M200). Nine or eleven reaction mixtures were prepared in a single experiment, and the time courses of the fluorescence were monitored simultaneously. A typical plate format is shown in Fig. 1A. After subtracting the background fluorescence, the fluorescence of the reaction mixtures was normalized to that of a reference sample containing 2.5% v/v seed solution and the same concentration of GuHCl, ThT, and MES but no monomer. Initial rates were determined by fitting an exponential or linear function to the initial region of each growth curve and taking the first derivatives of those functions at time zero. The experiments were performed in duplicate or triplicate, and the average and standard deviation values were presented in this work.

### Circular dichroism (CD) measurements

GuHCl-induced unfolding of monomeric PrP and the seed fibrils was examined at 37 °C using a Chirascan-plus CD spectrometer (Applied Photophysics) with a 1 mm path length quartz cuvette (Hellma, 100-QS). The concentration of monomeric PrP and the seed solution were 5 µM and 10% v/v, respectively, in the presence of 20 mM MES (pH 6) and various concentrations of GuHCl. CD spectra were recorded after 0 or 2 h of incubation at 37 °C. The unfolding curves for WT and V210A monomers were globally analyzed by a two-state unfolding model to derive a best fit value of ΔG.

$$\begin{array}{c}\theta ={n}_{i}\times \frac{1}{1+K}+({u}_{i}+{u}_{s}[GdHCl])\times \frac{K}{1+K}\\ K=\exp (\frac{-{\rm{\Delta }}G+m\times [GdHCl]}{RT})\end{array}$$

The best-fit values were as follows: 3.37 kcal/mol for ΔG of WT, 2.50 kcal/mol for ΔG of V210A, 2.00 kcal/mol/M for *m*, −6.74 mdeg for *n*_*i*_, −2.38 mdeg for *u*_*i*_, and 0.41 mdeg/M for *u*_*s*_.

### Electron microscopy (EM)

The seed solution was 10-fold diluted with deionized water and immediately loaded onto a carbon-coated grid (EM Japan, Cat. No. U1013). The grid was negatively stained with 2% phosphotungstate (pH 7) and examined using a transmission electron microscope (JEOL, JEM-2100 F).

## Electronic supplementary material

Supplementary Info
