# Supplementary material for: The native state of prion protein (PrP) directly inhibits formation of PrP-amyloid fibrils in vitro
Source: Sci Rep. 2017 Apr 3;7:562. doi: 10.1038/s41598-017-00710-x (PMC5429628; doi:10.1038/s41598-017-00710-x)
Supplement: Supplementary file 1 — Supplementary Info [file 41598_2017_710_MOESM1_ESM.pdf]

Supplemental information: the native state of prion protein (PrP) directly inhibits formation of PrP-amyloid fibrils in vitro

Ryo P. Honda and Kazuo Kuwata

Contents

1. Supplemental experimental procedures
2. Amyloid growth followed by size exclusion chromatography (SEC)
3. SEC and dynamic light scattering (DLS) experiments to probe the oligomerization of PrP
4. Comparison of *in situ* and *ex situ* assays
5. Unit conversion (RFU/% s^{-1} to s^{-1})
6. Supplemental figures (Figures S1–S8)
7. Supplemental references.
8. Supplemental tables (Tables S1 and S2)

1. Supplemental experimental procedures

Size exclusion chromatography (SEC). SEC experiments were performed using a TSKgel G3000SW column (TOSOH) on an AKTA purifier UPC10 system (GE healthcare). The running buffer contained the same concentrations of GuHCl and MES as the analyte solution. The flow rate was 1.0 mL/min at 25°C. For the experiments shown in Fig S2, 18 μM monomeric PrP was incubated with 10% v/v seed solution at 37°C in the presence of 20 mM MES (pH 6) and various concentrations of GuHCl. For the experiments shown in Fig S7, 18 or 150 μM monomeric PrP was incubated with the 2.5% v/v seed solution at 37°C in the presence of 20 mM MES (pH 6) and various concentrations of GuHCl. Aliquots of 45 μL were taken from the reaction mixtures at different points in the reaction period and directly injected into an analytical column. The elution profile was monitored by absorbance at 280 nm. The monomer consumption was calculated from the decrease in the peak area of the monomer.

Dynamic light scattering (DLS). The hydrodynamic properties of WT PrP were examined by DLS, at a protein concentration of 38–300 μM, in the presence of 20 mM MES (pH 6) and various concentrations of GuHCl. The viscosity of the protein solution was assumed to be identical to that of an aqueous solution of GuHCl (Kawahara and Tanford, 1966). Undesirable contaminants, such as dusts and bubbles, were removed from the protein solution by centrifugation at 15,000 rpm for 10 min at 4°C. More than 95% of proteins remained in the supernatant fraction, as judged by absorbance at 280 nm. The supernatant was analyzed using a Zetasizer Nano S (Malvern) at 37 °C with a disposable plastic micro cuvette (Malvern, Cat. No. ZEN0040). The volume distribution was derived from the first-order correlation function using software supplied by the manufacturer.

***Ex situ* ThT fluorescence measurements.** Monomers (18 or 150 µM) were incubated with 2.5% v/v seed solution at 37°C in the presence of 20 mM MES (pH 6) and various concentrations of GuHCl. Aliquots of 20 µL were taken from the reaction mixture at different times, and 10-fold diluted with a ThT solution containing 5 µM ThT, 20 mM MES (pH 6), and 2.0 M GuHCl. The fluorescence intensity at 485 nm (excitation at 445 nm) was immediately measured via fluorescence spectroscopy (Hitachi, F-7000) with a 5 × 5 mm^2 quartz cuvette. The fluorescence intensity was normalized to that of a reference sample containing 2.5% seed fibrils and the same concentration of GuHCl, ThT, and MES, but no monomer.

2. Amyloid growth followed by SEC.

Although the ThT assay has been established for quantitatively measuring the amount of amyloid fibrils, several lines of evidence suggest that ThT exhibits interference with amyloid growth or that ThT fluorescence is not directly proportional to the mass concentration of amyloid fibrils (Groenning, 2010; Saar et al., 2016). It has been also suggested that amyloid fibrils formed under different solution conditions exhibit differences in the intensity of ThT fluorescence. To test these possibilities, we utilized SEC to follow amyloid growth in the absence of ThT. We performed a seeded growth experiment in a ThT-free condition, in which 18 µM monomer were co-incubated with 10% seed fibrils under various concentrations of GuHCl. At different times in the reaction period, aliquots were taken from the reaction mixture and subsequently subjected to SEC analysis. As shown in Figures S2A and S2B, the peak area of monomer decreased as the reaction proceeded, indicating that the monomer was consumed and converted into amyloid fibrils. Importantly, when the rate of monomer consumption was compared with that of increase in ThT fluorescence, a good correlation was observed between the two variables (Figure S2C). This correlation demonstrated that the ThT has minimum interference with amyloid growth, and confirmed that our ThT assay quantitatively evaluated the growth reaction.

Another important finding in the SEC experiment was that the slope in the correlation plot of WT was identical (within the margin of error) to that of V210A (Figures S2C). A common slope of 1.5 relative fluorescence unit (RFU)/µM means that the conversion of 1 µM WT or V210A monomer to the amyloid fibril results in a 1.5 unit increase in ThT fluorescence. This indicates that an amyloid fibril formed by WT and V210A gives rise exactly to the same ThT fluorescence per weight of amyloid fibrils.

3. SEC and DLS experiments to probe the oligomerization of PrP.

A high concentration of PrP potentially induces the formation of an off-pathway oligomer (incapable of converting to fibrils) and subsequently produces an apparent decrease in the rate of amyloid formation (Souillac et al., 2002). To test this possibility, we performed a SEC experiment in which monomeric PrP at a high protein concentration (150 µM) was incubated with or without 2.5% seed fibrils for 2 h and subsequently subjected to SEC analysis. The result illustrated that the major fraction of PrP was eluted at the monomer position (8–10 mL), and the chromatograms displayed little or no sign of oligomerization (Figure S8A). To further validate this result, we performed a DLS experiment in which a volume distribution of WT PrP was examined at the various protein concentrations ranging from 38 to 300 µM. Under all the condition examined, WT PrP yields a monodisperse species with the hydrodynamic diameter of 6.1–7.5 nm (Figure S8B), which is in good agreement with a previously reported value for monomeric PrP (6–7 nm) (Martinez et al., 2015). Therefore, these two experiments consistently demonstrate that PrP can exist as a monomer even at high protein concentrations.

4. Comparison of *in situ* and *ex situ* assays.

Although it is generally assumed that ThT binding occurs more rapidly than amyloid formation, it remains unclear what occurs at a higher protein concentration where the amyloid formation becomes faster. If ThT binding is the rate-limiting step in the observed fluorescence changes, then we would underestimate the rate of amyloid formation. To test this possibility, we

performed a seeded growth experiment without ThT in the presence of a high and low concentration of monomer (150 and 18 µM, respectively). After various reaction times, 20 µL aliquots were withdrawn from the reaction mixture and directly added to a cuvette containing 180 µL of a ThT solution for the fluorescence measurement. The rationale behind this *ex situ* assay is that this method can measure the rate of amyloid growth independently of the rate of ThT binding. As shown in Figure S7, the *ex situ* assay yielded essentially the same growth rate as those measured by *in situ* assays in all conditions examined (Figure S9). We therefore concluded that the ThT binding occurs more rapidly than amyloid growth, and it is not a rate-limiting step in the observed fluorescence changes.

5. Unit conversion (RFU/% s^{-1} to s^{-1}).

To determine the absolute values for the rate constants of amyloid growth, it is necessary to convert the unit from RFU/% s^{-1} to s^{-1}. To this end, we first estimated the average polymerization degree of seed fibrils (*n*) by assuming a cylindrical shape as follows;

$$\mathrm{n} = \frac{\frac{(\pi(\frac{w}{2})^2 \times l)}{v} \times N_A}{MW}$$

In this equation, *w* and *l* are the width and length of the seed fibril, respectively, *v* is the specific volume of seed fibrils; N_A is Avogadro's number; *MW* is the molecular weight of the monomer (23 kDa). We estimated *w* and *l* as 13 and 122 nm, respectively, based on the electron microscopy observation (Figure 1B). If a specific volume of 1.3 g/cm^3 is assumed for the seed fibrils (Lee et al., 2009), a fibril of this size can contain up to 840 monomer molecules. This

value is consistent with the SEC experiment, in which the most fraction of seed fibrils was eluted in the void volume (>550 kDa, Figure S2A). In addition, a similar value of *n* was recently reported from an ultracentrifuge study of amyloid fibrils formed by β2-microglobulin (Chatani et al., 2009). They revealed that an amyloid fibril with the average polymerization degrees of 140–279 can be prepared using a serial PMCA protocol.

The next step was to calculate how many molecules of amyloid fibrils are present in the seed solution. Given that all monomers that were present in the seed solution (50 µM) were converted to amyloid fibrils with a polymerization degree of 840, the molar concentration of amyloid fibrils was expected to be 60 nM. We therefore estimated that 1% v/v of seed solutions contains approximately 0.60 nM of amyloid fibrils (*i.e.*, 1% = 0.60 nM). Here, the number of growth ends of individual fibrils was assumed to be one for simplicity. Combined with the fact that a structural conversion of 0.7 µM monomer into amyloid fibril yields an increase in one RFU of ThT fluorescence (*i.e.*, 1 RFU = 0.7 µM) (see Figure S2C), we obtained the unit conversion factor of 1 RFU/% $s^{-1} = 1.2 \times 10^{3} s^{-1}$.

6. Supplemental Figures

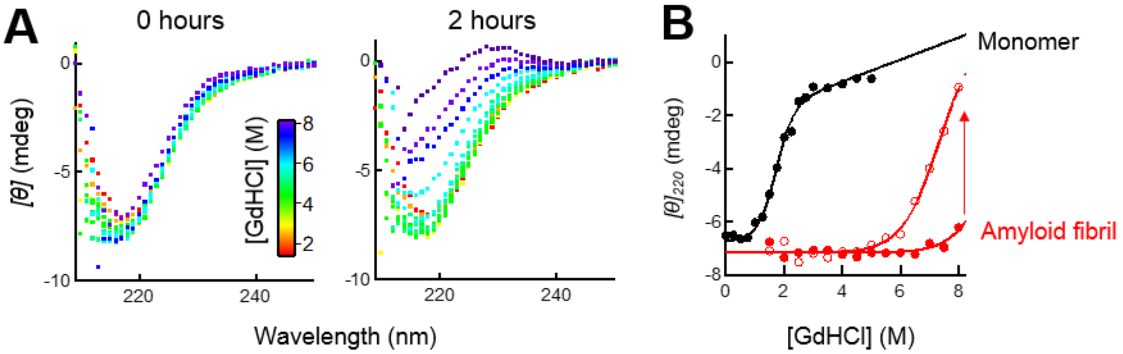

Figure S1. (A) Far-UV CD spectra for seed fibrils in different concentrations of GuHCl. The CD spectra were acquired after the 0 (left) and 2 (right) h of incubation at 37°C. The concentration of seed fibrils was 10%. (B) GuHCl-unfolding curves for WT monomer (black circles), and amyloid fibrils with (empty red circles) or without (filled red circles) 2 h of incubation at 37°C. The solid lines are shown only to guide the eye.

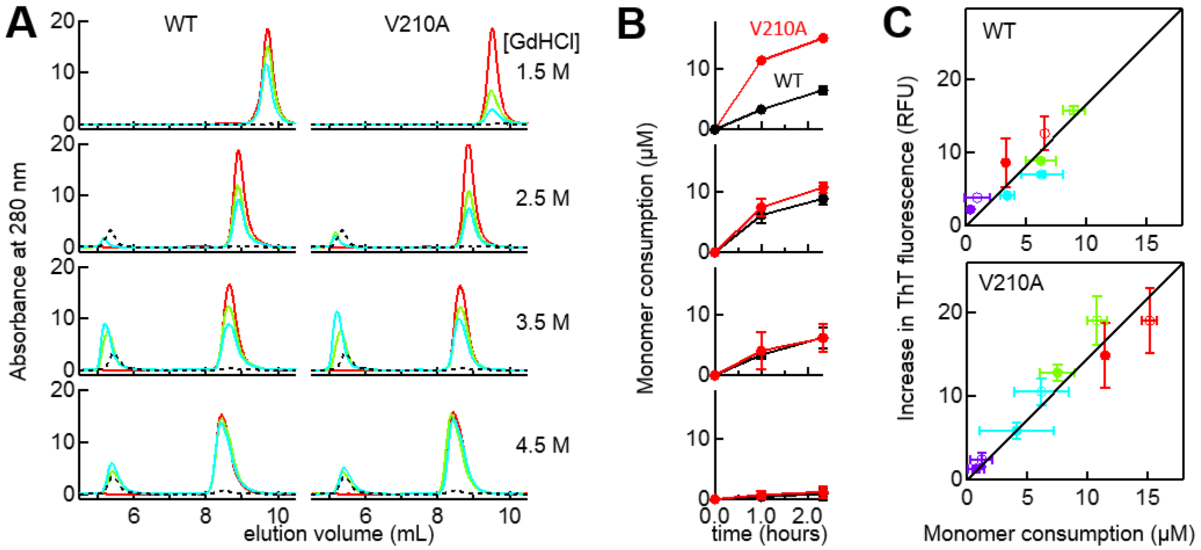

Fig S2. Monomer consumption during the seeded growth reaction. (A) Elution profile (WT [left column] and V210A [right column]) at different times (1.0 [light green] and 2.3 h [light blue]). Elution profiles for monomer only (red) and seed fibrils only (dotted black) are also shown. Of note, the peak of seed fibrils (5–6 mL) was less pronounced under low GuHCl concentration, such as 1.5 M, probably due to the interaction between the seed fibrils and the column. (B) Time courses of monomer consumption (WT [black] and V210A [red]) at various concentrations of GuHCl (from 1.5 to 4.5 M, from top to bottom). (C) A correlation between monomer consumption and the increase in ThT fluorescence during the growth reaction (1.5 M [red], 2.5 M [light green], 3.5 M [light blue], and 4.5 M GuHCl [purple]). The filled and empty circles represent the values at 1.0 and 2.3 h, respectively. The increase in ThT fluorescence was derived from Figures S3 and S4. The correlation coefficients between the two variables were 0.86 for WT and 0.96 for V210A, and the slopes in the correlation plots are 1.64 ± 0.16 RFU/μM, and 1.45 ± 0.09 RFU/μM.

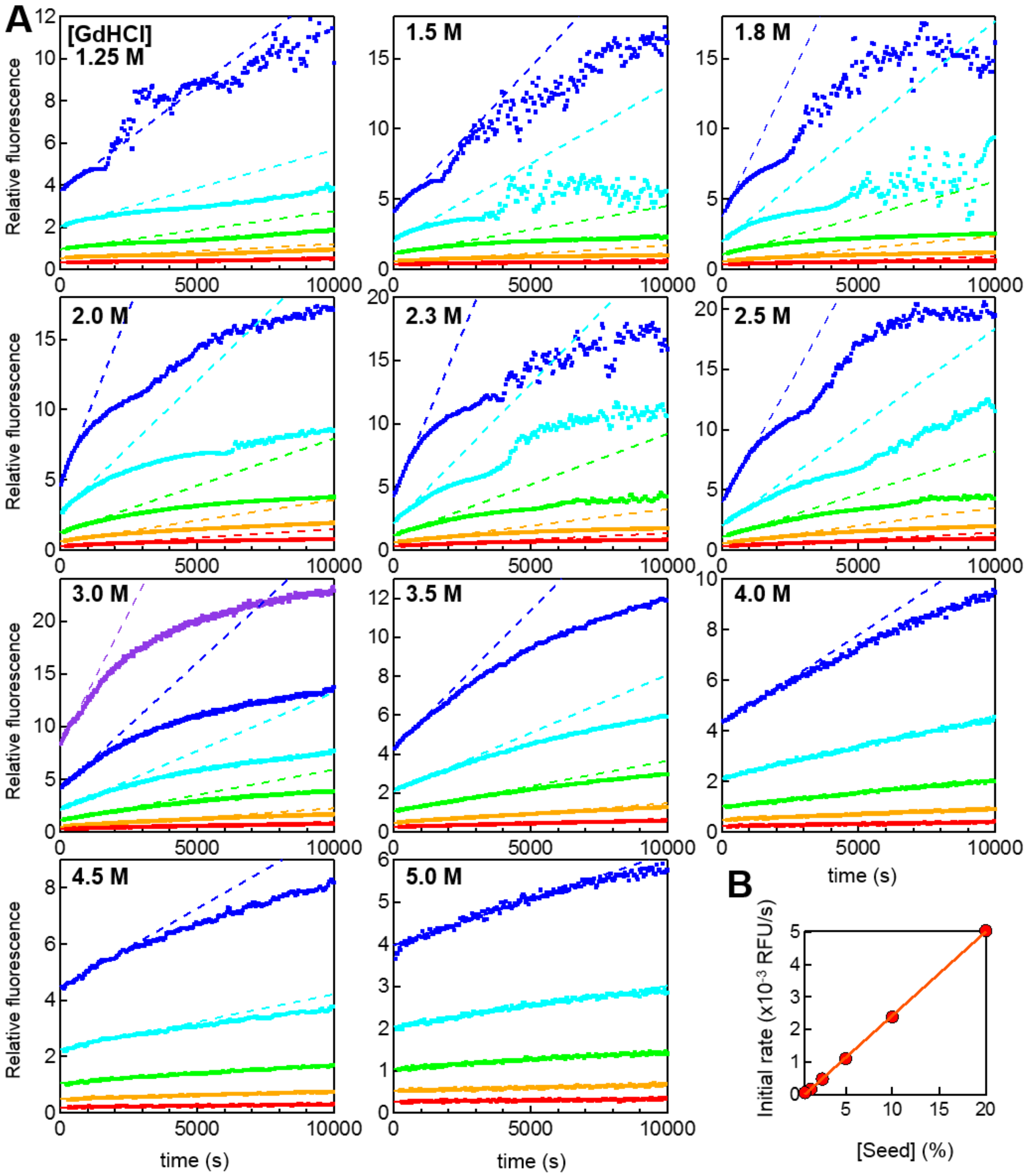

Fig S3. (A) Amyloid growth reaction of WT PrP when the concentration of seed fibrils is varied. The reaction was monitored by the change in ThT fluorescence in the presence of different concentrations of seeds (0.6% [red], 1.3% [orange], 2.5% [green], 5.0% [light blue], 10.0% [dark

blue], and 20.0% [purple]). The initial concentration of monomeric PrP was 18 µM. The GuHCl concentration of the reaction mixture is shown in the top left corner of each image. The dotted lines represent the linear fits to the initial increase in fluorescence. (B) Initial rates at 3.0 M GuHCl as a function of the seed concentration. Note that a linear correlation exists up to 20%.

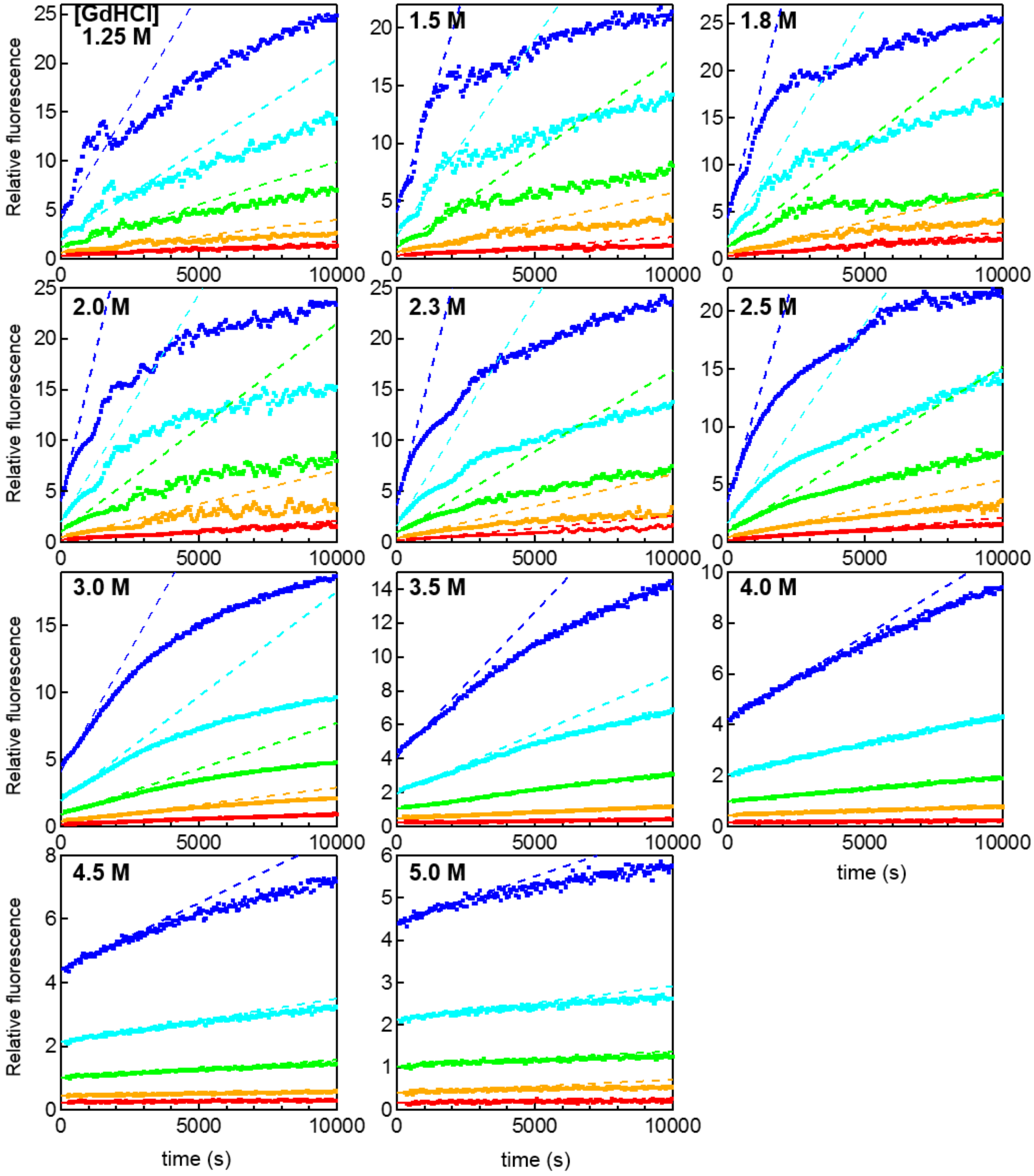

Fig S4. Fibril growth reaction of V210A when the concentration of seed is varied. The reactions were seeded with the different concentrations of WT PrP fibrils seeds (0.6% [red],

1.3% [orange], 2.5% [green], 5.0% [light blue], and 10.0% [dark blue]), and monitored by the change in ThT fluorescence. The initial concentration of monomeric PrP was 18 µM. The dotted lines represent the linear fits to the initial increase in fluorescence.

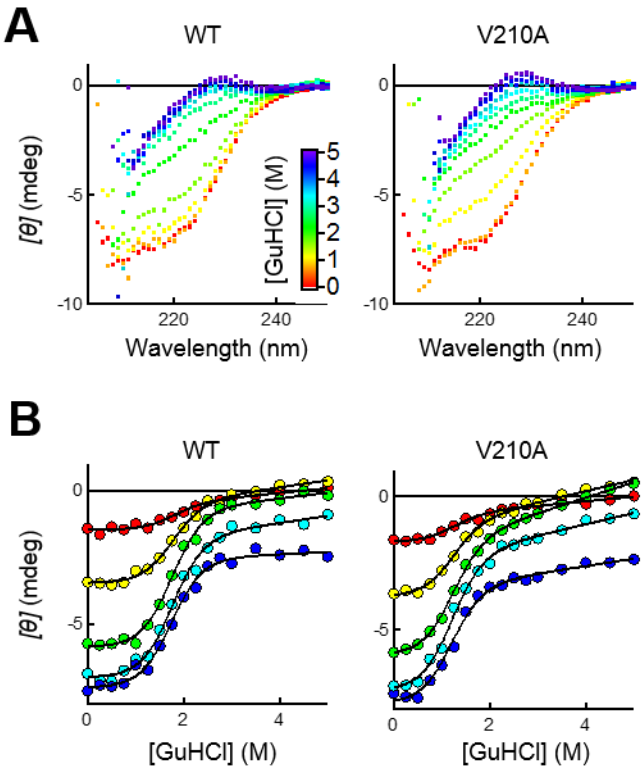

Figure S5. (A) Far-UV CD spectra for monomeric PrP in different concentrations of GuHCl (WT [left panel] and V210A [right panel]). (B) GuHCl-unfolding curves at selected wavelengths (WT [left panel] and V210A [right panel]): 235 (red), 230 (yellow), 225(green), 220 (light blue) and 215 nm (dark blue). The lines represent the best global fit of a two-state unfolding model where two parameters (ΔG and m) were used as global fitting parameters.

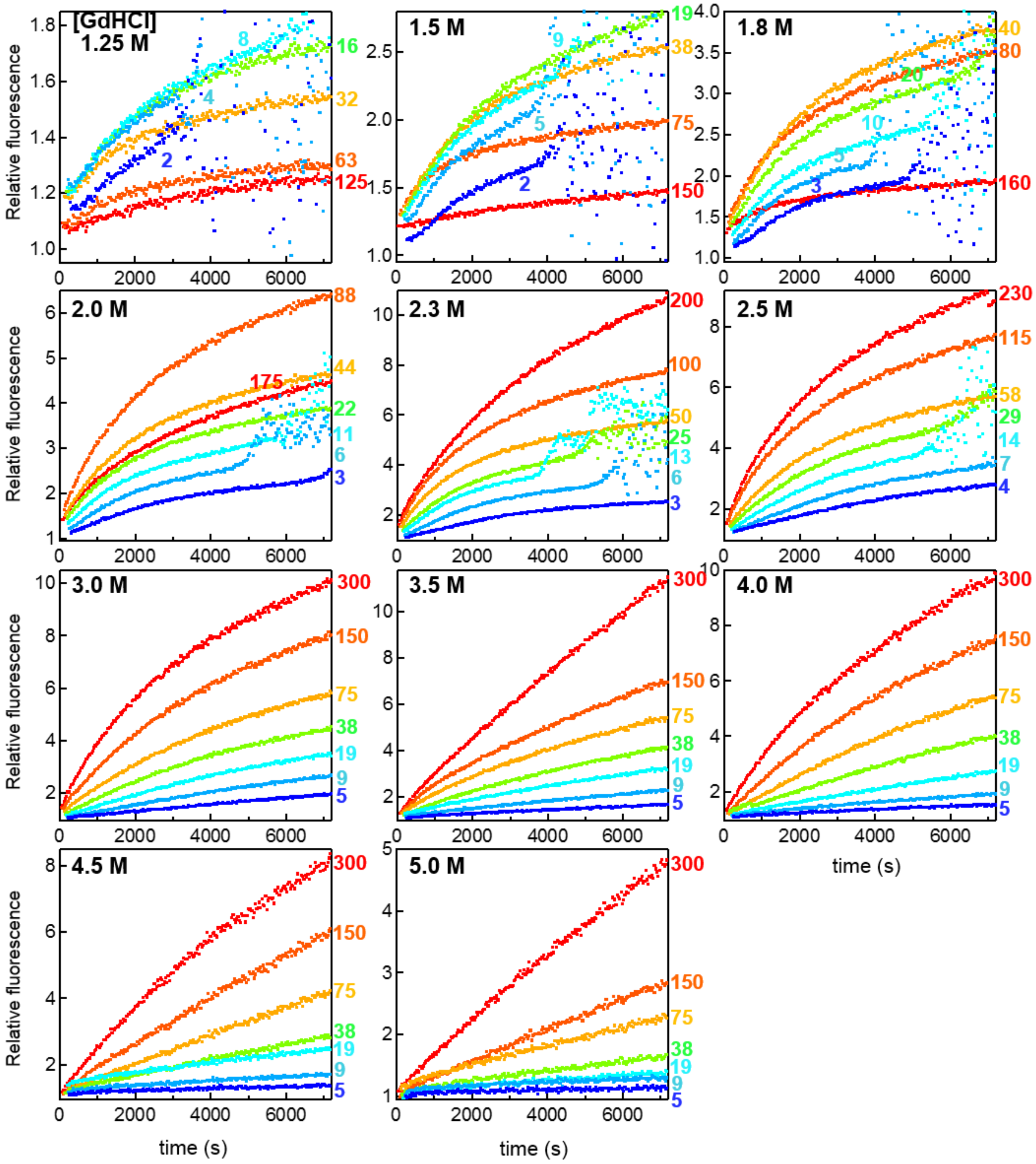

Fig S6. Amyloid growth of WT PrP when the concentration of monomer is varied. The initial concentrations of monomer are shown next to the curves in the unit of µM. The reactions were seeded with 2.5% WT PrP fibrils.

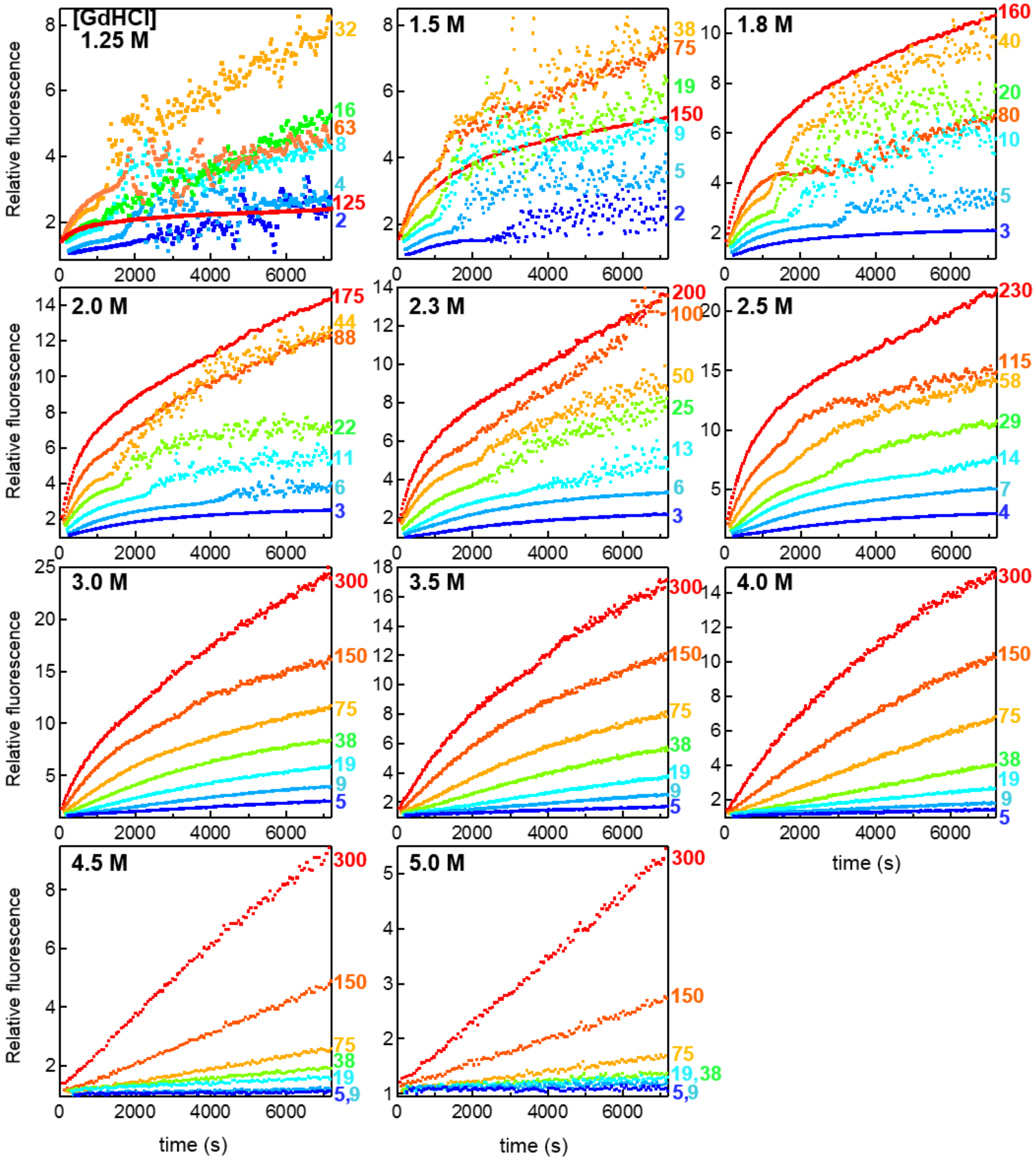

Fig S7. Amyloid growth of V210A PrP when the concentration of monomer is varied. The initial concentrations of monomer are shown next to the curves in the unit of µM. The reactions were seeded with 2.5% WT PrP fibrils.

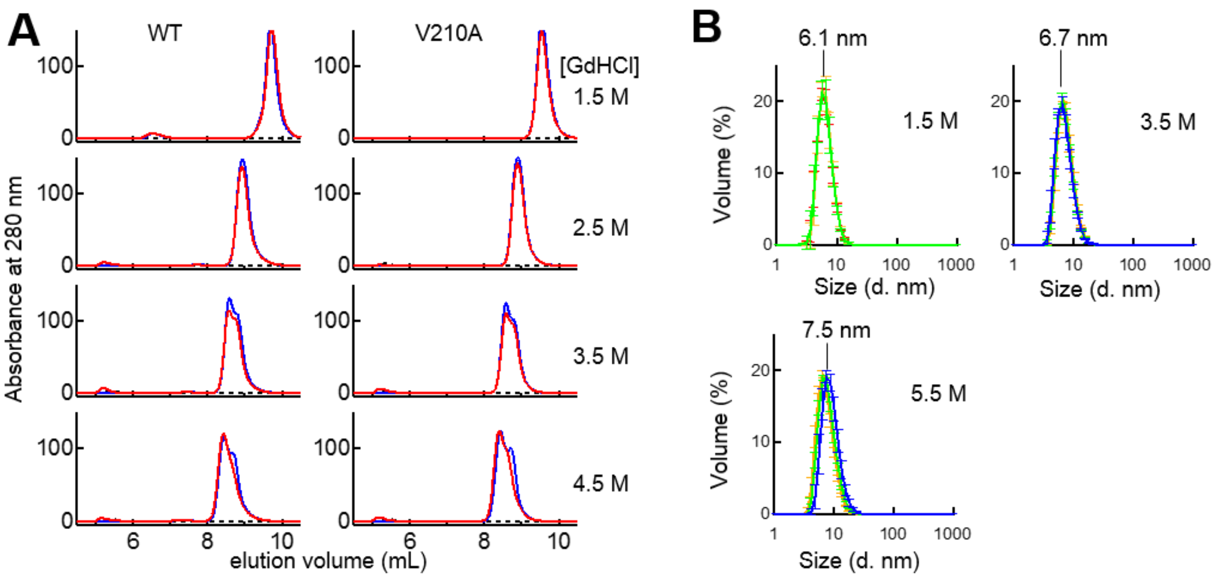

Fig S8. Size exclusion chromatography (SEC) and dynamic light scattering (DLS) experiments. (A) Elution profiles for WT (left column) and V210A (right column). The analyte solution contained 150 µM monomeric PrP with (red) or without (blue) 2.5% seed fibrils. (B) Volume distribution of WT PrP. The data were obtained at 37°C in the presence of different concentrations of GuHCl (1.5, 3.5, and 5.5 M) and different concentrations of PrP (38 [red], 75 [orange], 150 [green], and 300 µM [blue]).

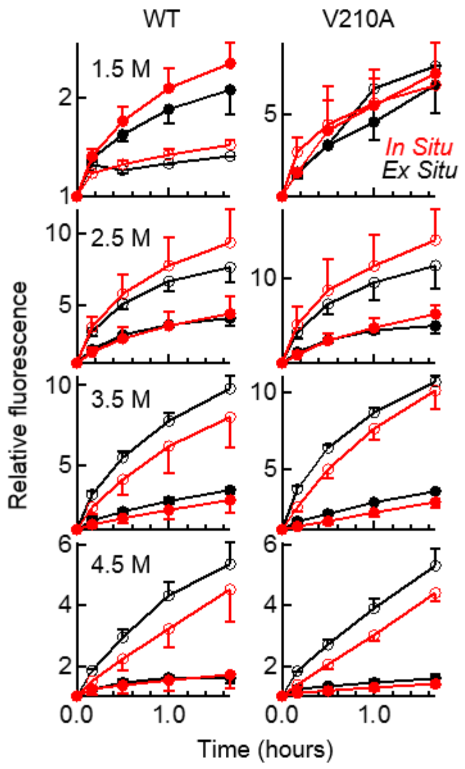

Fig S9. Comparison of *in situ* and *ex situ* assays. Time courses of ThT fluoresecne during the growth reaction as measured by the *in situ* (red symbols) or the *ex situ* assay (black symbols). The reaction mixtures contained 18 (filled symbols) or 150 µM (empty symbols) monomeric PrP (WT [left column] and V210A [right column]) and 2.5% seed fibrils in the presence of various concentrations of GuHCl (1.5–4.5 M, from top to bottom). The values for the *in situ* assay were derived from Figure S6 or S7.

7. Supplementral Reference

Chatani, E., Lee, Y.H., Yagi, H., Yoshimura, Y., Naiki, H., and Goto, Y. (2009). Ultrasonication-dependent production and breakdown lead to minimum-sized amyloid fibrils. Proc Natl Acad Sci U S A *106*, 11119-11124.

Groenning, M. (2010). Binding mode of Thioflavin T and other molecular probes in the context of amyloid fibrils-current status. Journal of chemical biology *3*, 1-18.

Kawahara, K., and Tanford, C. (1966). Viscosity and density of aqueous solutions of urea and guanidine hydrochloride. Journal of Biological Chemistry *241*, 3228-3232.

Lee, Y.-H., Chatani, E., Sasahara, K., Naiki, H., and Goto, Y. (2009). A comprehensive model for packing and hydration for amyloid fibrils of β2-microglobulin. Journal of Biological Chemistry *284*, 2169-2175.

Martinez, J., Sanchez, R., Castellanos, M., Makarava, N., Aguzzi, A., Baskakov, I.V., and Gasset, M. (2015). PrP charge structure encodes interdomain interactions. Sci Rep *5*, 13623.

Saar, K.-L., Yates, E.V., Müller, T., Saunier, S., Dobson, C.M., and Knowles, T.P. (2016). Automated Ex Situ Assays of Amyloid Formation on a Microfluidic Platform. Biophys J *110*, 555-560.

Souillac, P.O., Uversky, V.N., Millett, I.S., Khurana, R., Doniach, S., and Fink, A.L. (2002). Effect of association state and conformational stability on the kinetics of immunoglobulin light chain amyloid fibril formation at physiological pH. The Journal of biological chemistry *277*, 12657-12665.

8. Supplemental tables

Supplemental table 1. Elementary constants for the growth reaction of WT PrP (note: 1 RFU/% ≈ 1.2 × 10^3).

[GuHCl] (M)	model	k_2 ($\times 10^{-3}$ RFU/% s^{-1})	K_m (μM)	k_2/K_m ($\times$ 10 RFU/% M^{-1} s^{-1})	K_i (μM)	f_N[5]	f_U[5]
0	-	0.75[4]	0.87[4]	86.2[4]	7.87×10^{-3} [4]<br>[1.94×10^{-2}][4]	0.99	0.01
1.25	two-step + uncomp[2]<br>[two-step + noncomp[3]]	(0.97)[6]	6.43 ± 0.94<br>[3.53 ± 2.55]	15.1 ± 2.2<br>[27.5 ± 19.9]	2.31 ± 0.33<br>[3.66 ± 2.45]	0.72	0.23
1.50	two-step + uncomp[2]<br>[two-step + noncomp[3]]	(1.02)[6]	9.43 ± 1.61<br>[6.19 ± 4.44]	10.8 ± 1.9<br>[16.5 ± 11.8]	5.22 ± 0.88<br>[7.14 ± 4.97]	0.63	0.37
1.75	two-step + uncomp[2]<br>[two-step + noncomp[3]]	(1.08)[6]	6.27 ± 1.54<br>[5.37 ± 1.72]	17.2 ± 4.2<br>[20.1 ± 6.4]	18.5 ± 4.6<br>[20.8 ± 6.77]	0.45	0.55
2.00	two-step + uncomp[2]<br>[two-step + noncomp[3]]	(1.13)[6]	12.9 ± 2.3<br>[12.2 ± 6.8]	8.76 ± 1.56<br>[9.26 ± 5.16]	80.0 ± 24.1<br>[88.4 ± 35.8]	0.28	0.72
2.25	two-step[1]	1.09 ± 0.06	24.9 ± 4.7	4.38 ± 0.86	N.D.	0.16	0.84
2.50	two-step[1]	1.50 ± 0.12	60.3 ± 12	2.49 ± 0.55	N.D.	0.09	0.91
3.00	two-step[1]	1.88 ± 0.00	158 ± 22	1.19 ± 0.16	N.D.	0.02	0.98
3.50	two-step[1]	1.12 ± 0.05	134 ± 13	0.84 ± 0.09	N.D.	0.01	0.99
4.00	two-step[1]	1.54 ± 0.06	334 ± 20	0.46 ± 0.03	N.D.	0.00	1.00
4.50	two-step[1]	N.D.	>500	0.21 ± 0.00	N.D.	0.00	1.00
5.00	two-step[1]	N.D.	>500	0.10 ± 0.00	N.D.	0.00	1.00

[1]Two-step reaction model (Figure 2D). [2]Uncompetitive model (Figure 5A). [3]Noncompetitive model (Figure 5B). [4]These values were estimated via linear extrapolation of log(X) vs. GuHCl concentration plot. [5]Fraction of native or unfolded state determined from the far-UV CD experiments (Figure 2C). [6]These values were estimated via linear extrapolation of log(k_2) vs. GuHCl concentration plot (Figure 3B) and fixed in the least-squares fitting. N.D., not determined. Error, the fitting error in one SD.

Supplemental table 2. Elementary constants for the growth reaction of V210A PrP (note: 1 RFU/% ≈ 1.2 × 10^3).

[GuHCl] (M)	model	k_2 (× 10^{-3} RFU/% s^{-1})	K_m (μM)	k_2/K_m (× 10 RFU/% M^{-1} s^{-1})	K_i (μM)	f_N[5]	f_U[5]
0	-	3.23[4]	3.91[4]	82.6[4]	3.96×10^{-3} [4]<br>[1.16×10^{-2}][4]	0.97	0.03
1.25	two-step + uncomp[2]<br>[two-step + noncomp[3]]	(4.16)[6]	40.8 ± 6.9<br>[31.2 ± 7.3]	10.2 ± 1.7<br>[13.3 ± 3.1]	15.2 ± 3.0<br>[23.6 ± 6.6]	0.47	0.53
1.50	two-step + uncomp[2]<br>[two-step + noncomp[3]]	(4.40)[6]	35.3 ± 4.7<br>[31.0 ± 6.6]	12.5 ± 1.7<br>[14.2 ± 3.0]	33.0 ± 6.3<br>[43.6 ± 9.8]	0.30	0.70
1.75	two-step[1]	4.07 ± 0.21	61.4 ± 7.6	6.63 ± 0.89	N.D.	0.17	0.83
2.00	two-step[1]	3.99 ± 0.19	53.4 ± 6.0	7.47 ± 0.91	N.D.	0.09	0.91
2.25	two-step[1]	5.08 ± 0.06	109 ± 11	4.66 ± 0.47	N.D.	0.05	0.95
2.50	two-step[1]	6.20 ± 0.36	163 ± 18	3.80 ± 0.47	N.D.	0.02	0.98
3.00	two-step[1]	7.08 ± 0.42	367 ± 33	1.93 ± 0.21	N.D.	0.01	0.99
3.50	two-step[1]	N.D.	>500	0.68 ± 0.01	N.D.	0.00	1.00
4.00	two-step[1]	N.D.	>500	0.42 ± 0.00	N.D.	0.00	1.00
4.50	two-step[1]	N.D.	>500	0.20 ± 0.01	N.D.	0.00	1.00
5.00	two-step[1]	N.D.	>500	0.08 ± 0.00	N.D.	0.00	1.00

[1]Two-step reaction model (Figure 2D). [2]Uncompetitive model (Figure 5A). [3]Noncompetitive model (Figure 5B). [4]These values were estimated via linear extrapolation of log(X) vs. GuHCl concentration plot. [5]Fraction of native or unfolded state determined from the far-UV CD experiments (Figure 2C). [6]These values were estimated via linear extrapolation of log(k_2) vs. GuHCl concentration plot (Figure 3B) and fixed in the least-squares fitting. N.D., not determined. Error, the fitting error in one SD.
